# Membrane-bound and soluble forms of an NMDA receptor extracellular domain retain epitopes targeted in auto-immune encephalitis

**DOI:** 10.1186/s12896-018-0450-1

**Published:** 2018-06-27

**Authors:** Rashmi Sharma, Fetweh H. Al-Saleem, Rama Devudu Puligedda, Amy Rattelle, David R. Lynch, Scott K. Dessain

**Affiliations:** 1 Lankenau Institute for Medical Research, 100 E. Lancaster Ave, Wynnewood, PA 19096 USA; 2 Division of Neurology, Children’s Hospital of Pennsylvania, Philadelphia, PA 19104 USA

**Keywords:** Anti-N-methyl-D-aspartate receptor encephalitis, ANRE, NMDA receptor, Monoclonal antibody, Autoimmunity, Antigen, TEV protease, Conformational epitope, Recombinant protein expression, Autoimmune encephalitis

## Abstract

**Background:**

Anti-NMDA receptor encephalitis (ANRE) is a potentially lethal disease attributed to auto-antibodies against the N-methyl-D-aspartate receptor (NMDAR). Full recovery is possible if therapy is initiated early in the disease course. Detection of ANRE antibodies in the cerebrospinal fluid (CSF) is essential for diagnosis. The assays for ANRE-associated IgGs often rely on cells transiently transfected with NMDAR genes. A cell line that stably expresses pathogenic NMDAR epitopes could improve standardization of the assays and provide antigen that could be used in commercial solid state assay systems.

**Results:**

We expressed the amino terminal domain (ATD) of the GluN1 NMDAR subunit (NR1) as a fusion protein on the outer plasma membrane of 293T cells, creating a stable cell population (293T-ATD) that is recognized by ANRE patient monoclonal antibodies in flow cytometry and immunofluorescence assays. The ATD fusion protein also contains a Myc tag and a 6XHIS tag, which provide functionality for immunoassays and antigen purification, and a TEV protease site, which allows the ATD domain to be specifically released from the cells in essentially pure form. ATD mobilized from the 293T ATD cell line maintained the pathogenic ANRE epitopes in ELISA binding assays. CSF (3/4) and sera (4/4) from ANRE patients also bound the 293T-ATD cell line, whereas normal CSF and sera did not.

**Conclusions:**

The 293T-ATD cell line is potentially adaptable to a variety of formats to identify antibodies associated with ANRE, including cell-based and soluble antigen formats, and demonstrates a useful method to produce complex proteins for research, drug discovery, and clinical diagnosis.

**Electronic supplementary material:**

The online version of this article (10.1186/s12896-018-0450-1) contains supplementary material, which is available to authorized users.

## Background

Anti-N-methyl-D-aspartate Receptor Encephalitis (ANRE) is an autoimmune syndrome that results from autoantibodies targeting the GluN1 subunit of the NMDA receptor (NR1) in the hippocampus and cortex [1, 2]. Patients with ANRE exhibit heterogeneous psychiatric and neurologic symptoms, which include memory loss, psychosis, hallucinations, seizures, autonomic nervous system dysfunction and catatonia [3, 4]. The symptoms of the disease may result from IgG-induced down-modulation of NMDA clusters and synaptic currents in hippocampal post-synaptic dendrites [5, 6]. ANRE is the most common of an expanding list of autoimmune encephalitis syndromes mediated by antibodies against cell surface or synaptic proteins [7].

Full recovery from ANRE is possible, but early diagnosis and treatment are essential [8]. Treatment includes therapies to reduce anti-NR1 antibody titers in the CSF and surgical removal of ovarian teratomas, which are associated with the disease in some cases [4]. However, diagnostic testing for anti-NR1 antibodies can be technically challenging, especially for assessing anti-NMDAR IgGs in patient sera [8, 9]. This is in part because the pathogenic epitopes include post-translational modifications that only occur in mammalian cells, and over-expression of the native NMDAR can be toxic to cultured cells [10]. Consequently, current Cell Based Assays (CBA) and ELISAs rely on transiently transfected cells [8]. A stable cell line that replicated authentic pathogenic NMDAR epitopes could improve standardization of the assay, as well as provide antigen that could be used in commercial solid state assay systems.

ANRE IgGs recognize the NR1 subunit within its extracellular amino-terminal domain (ATD), which binds the co-agonist glycine and regulates NR1 ion channel function [11, 12]. The ATD of NR1 is both necessary and sufficient for staining by ANRE patient antibodies [10]. The region required for NR1 binding to ANRE IgG includes amino acids N368 and G369, which mediate post-translational modifications critical for IgG binding [10]. We previously studied a mutant NR1 that contained only the ATD and the C-terminal transmembrane domain. In this study, we stably expressed the NR1 ATD on the outer plasma membrane of 293T cells, as a fusion protein that contains a Myc tag, a 6XHIS tag, a TEV protease site, and the PDGF receptor transmembrane domain. We assessed ATD binding in cell based assays and ELISAs with a commercial NR1 mAb, ANRE patient CSF, three human anti-NR1 IgG mAbs from an ANRE patient, and an additional panel of ANRE and normal patient sera and CSF samples.

## Results

### Expression of an ATD fusion protein on the surface of 293T cells

**Fig. 1:**
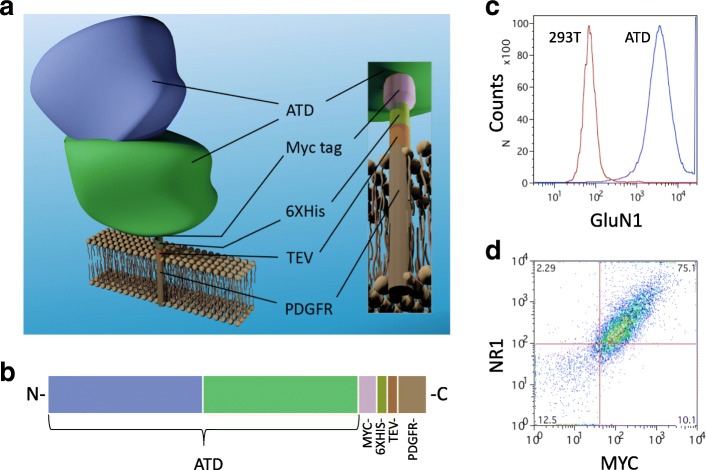
Structure and expression of the NR1 Amino Terminal Domain (ATD) fusion protein on 293 T cells. (**a, b**) The ATD fusion protein consists of the entire 561 N-terminal amino acid extracellular domain, which includes the bi-lobed NR1 ATD, fused in sequence to the Myc tag, the 6XHIS tag, the Tobacco Etch Virus (TEV) protease site, and the platelet derived growth factor receptor (PDGFR) transmembrane domain. The cartoon is not strictly drawn to scale. Color code: blue and green; extracellular GluN1 bi-lobed domain; purple, Myc tag; light green, 6XHIS tag; orange, TeV protease site; brown, PDGFR transmembrane domain. (**c, d**) Expression of the ATD fusion protein on the surface of 293T cells was analyzed by flow cytometry with a commercial anti-GluN1 mAb either alone (**c**) or with an anti-Myc tag mAb (**d**)

We designed a recombinant gene encoding the first 561 amino acids of NR1, the Myc epitope tag (EIDSEEKL), a 6XHIS tag, and the Tobacco Etch Virus (TEV) protease cleavage site (ENLYFQGG), fused to the platelet derived growth factor receptor (PDGFR) transmembrane domain (Fig. 1a-b) [13]. We used retroviral transduction to express the gene in 293T cells under puromycin selection. A stable polyclonal population was isolated by flow cytometry over four rounds of selection using a commercial murine NR1 mAb, resulting in the cell population 293T-ATD (Fig. 1c). Co-staining of the cell population with a Myc tag antibody and the NR1 mAb indicates that most of cells that express the Myc tag also express the NR1 ATD (Fig. 1d).

**Fig. 2:**
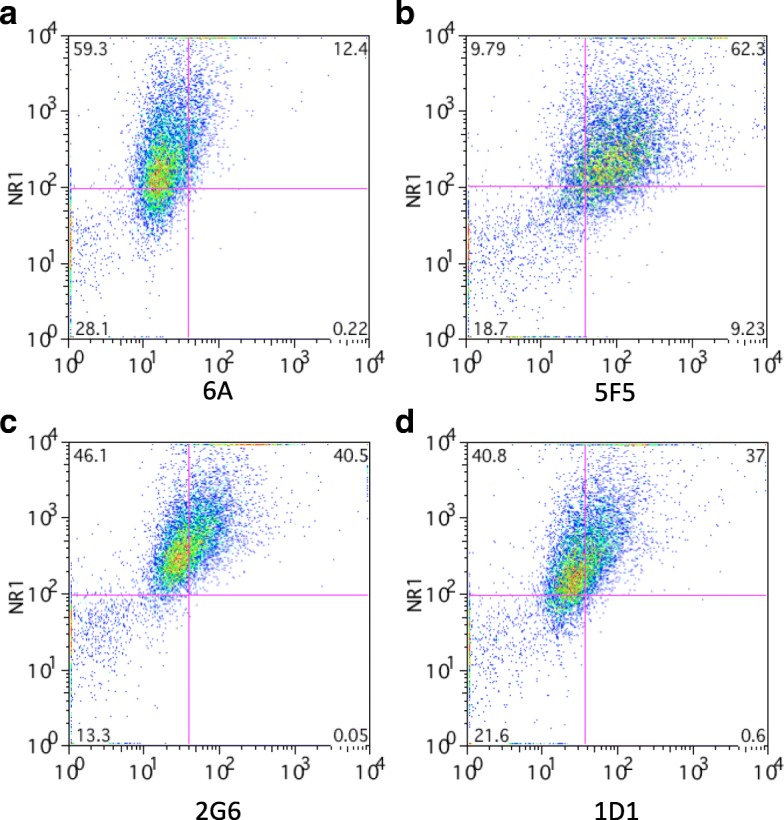
Binding of human ANRE mAbs to 293T-ATD cells by flow cytometry. Cells were immunostained with a commercial anti-NR1 mAb and a human mAb and analyzed by flow cytometry. Human mAbs were either the isotype control IgG 6A (**a**), or ANRE patient mAbs 5F5 (**b**), 2G6 (**c**), or 1D1 (**d**)

We previously cloned three human monoclonal antibodies (mAbs), 5F5, 2G6, and 1D1, from a female ANRE patient (*manuscript in press*). We tested binding of the mAbs to the 293T-ATD cell population using flow cytometry, co-staining with the commercial anti-NR1 mAb (Fig. 2). The ANRE patient mAbs all bound to the 293T-ATD cells to a greater extent than the 6A isotype control mAb, with double positive cells comprising 62.3% (5F5), 40.5% (2G6), and 37.0% (1D1), compared to 12.4% (6A control), Calculated as the proportion of NR1 positive cells bound by the mAbs, the 5F5 showed 90.5% binding; 2G6, 58.8%; 1G1, 53.7%; and 6A, 18.0% (Table 1).

**Table 1:** Percent GluN1-expressing cells bound by human ANRE mAbs

Human mAb	Percent GluN1 positive 293T-ATD cells bound
5F5	90.5
2G6	58.8
1D1	53.7
6A	18.0

### Immunofluorescence detection of NR1-antibody binding to 293T-ATD cells

**Fig. 3:**
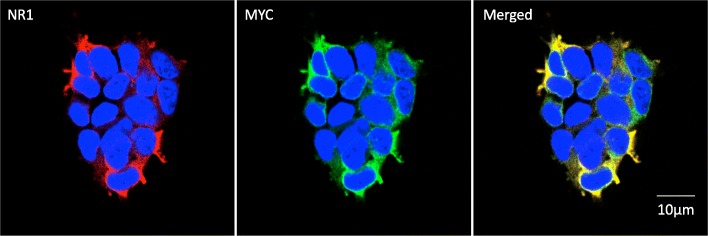
Immunofluorescence imaging of the NR1 ATD fusion protein on 293T-ATD cells. 293T-ATD cells were immunostained with a murine anti-NR1 mAb (red color, left panel) and the anti-Myc-tag mAb (green color, middle panel). A merged image is also shown (right panel). Nuclei were stained with DAPI, and the cells were visualized by confocal microscopy. Scale bar = 10 μm

**Fig. 4:**
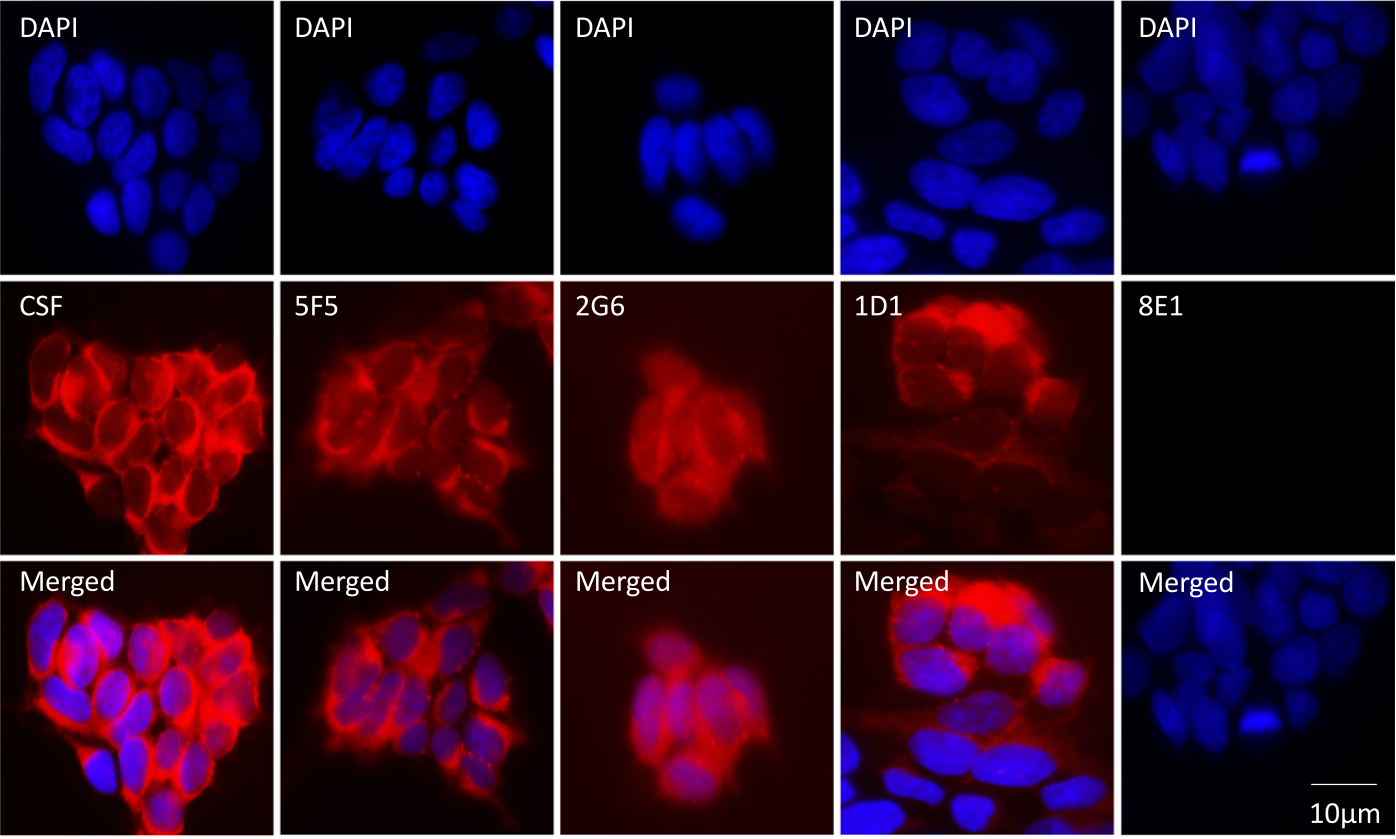
Binding of human ANRE patient CSF and mAbs to 293T-ATD cells by immunofluorescence. 293T-ATD cells were stained with ANRE patient CSF, human ANRE mAbs 5F5, 2G6, and 1D1, or the 8E1 isotype control mAb (red). Nuclei were stained with DAPI (blue) and the cells were visualized by confocal microscopy. Scale bar = 10 μm

We next tested the 293T-ATD cell line for detecting NR1 antibodies by immunofluorescence. We first co-stained the cells with the commercial NR1 and Myc antibodies, and observed co-localization of the signals on the outer plasma membrane (Fig. 3). We then tested binding of ANRE patient CSF, the three ANRE patient mAbs, and an isotype control human mAb, 8E1 (Fig. 4). CSF and the ANRE patient mAbs all reacted with the 293 T-ATD cell line, whereas the 8E1 mAb did not.

### ELISA studies of TEV protease-mobilized ATD

**Fig. 5:**
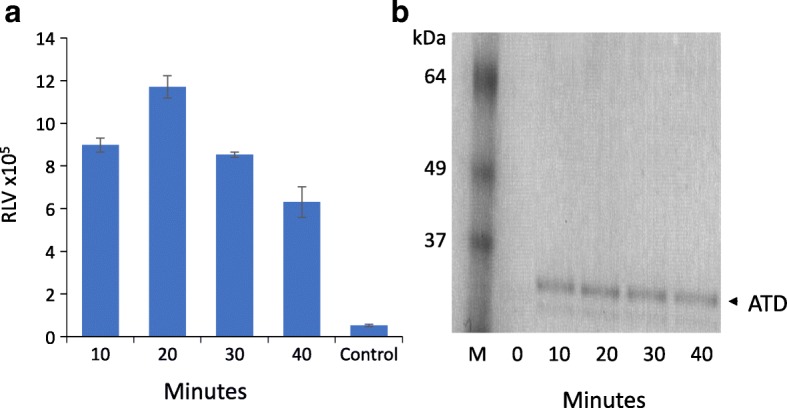
Mobilization of membrane-bound ATD with TEV protease. 293T-ATD cells were washed and then treated with TEV protease for 10, 20, 30, or 40 min. Expressed protein was analyzed by (**a**) capture ELISA and (**b**) Coomassie-stained SDS:PAGE. ATD, amino-terminal domain; Control, medium-only blank; M, marker; RLV, relative luminescence value. Bars indicate the S.E.M.

The TEV protease site adjacent to the PDGF transmembrane domain was included to allow mobilization of the expressed ATD for use in binding studies requiring soluble antigen. We treated the 293T-ATD cells with TEV protease for 10–40 min, spun down the cells, and analyzed the reaction supernatants by capture ELISA and Coomassie-stained SDS:PAGE (Fig. 5). Analyzed by ELISA, the ATD was evident at 10 min and peaked at 20 min, and declined somewhat thereafter (Fig. 5a). Longer incubations (up to 2 h) futher decreased amount of mobilized ATD (data not shown). The non-denaturing SDS:PAGE gel gave a dominant band at approximately 25 kDa, with a faint band slightly below, and no significant bands above, demonstrating the specificity of cleavage of the ATD from the outer plasma membrane.

**Fig. 6:**
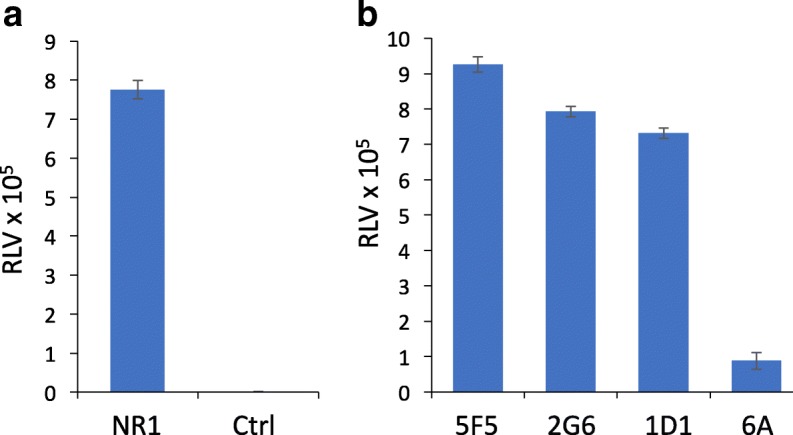
Binding of human anti-NR1 mAbs to plate-adherent ATD. ATD mobilized by TEV protease treatment of 293T-ATD cells was captured by a Myc tag antibody and tested for binding by commercial and human mAbs. **a** Murine anti-NR1 mAb. **b** Human mAbs, 5F5, 2G6, 1D1, and 6A (isotype control). All samples were tested in triplicate. RLV, relative luminescence value; Ctrl, buffer only. Bars indicate the S.E.M.

**Fig. 7:**
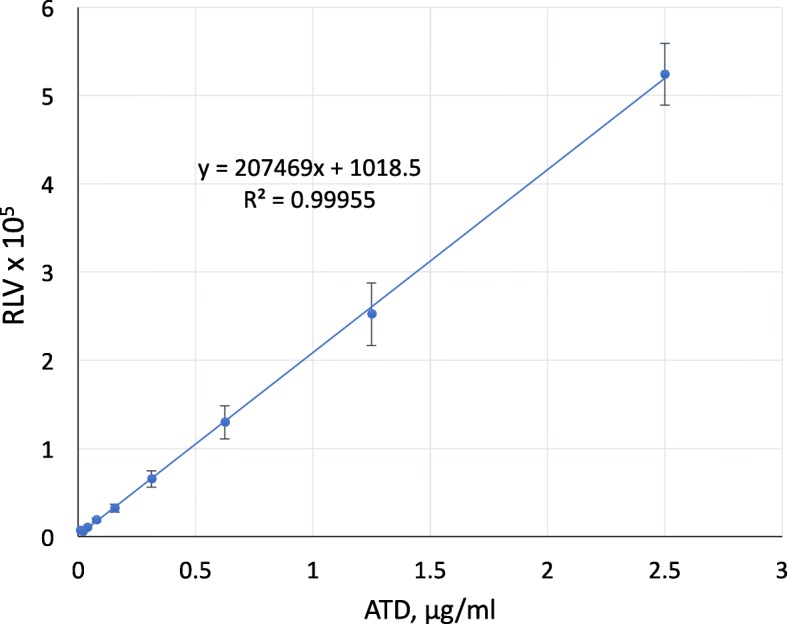
Titration of ATD protein in a capture ELISA. A titration of TEV-mobilized ATD was tested for binding to plate-adherent 5F5 mAb. ATD was biotinylated and tested from 65 pg/ml to 5 μg/ml, in triplicate samples, and detected with SA-HRP. The relative luminescence signal was measured. Calculated R2 = 0.99955. RLV, relative luminescence value. Bars indicate the S.E.M.

We next tested antibody binding to the mobilized ATD in a capture ELISA format. We used a Myc tag antibody to capture the ATD onto ELISA plates and tested binding of the commercial NR1 and human ANRE mAbs. The murine NR1 mAb bound significantly above background levels (Fig. 6a). Similarly, the three human ANRE mAbs all bound the plate-adherent ATD, giving signals approximately 8–10 fold greater than the 6A human isotype control mAb (Fig. 6b). We next tested whether the ATD could be used reproducibly in a quantitative assay. We biotinylated the ATD, then tested its binding to plate-immobilized 5F5 antibody in an ELISA, using SA-HRP for detection. We tested triplicate samples ranging from 65 pg/ml to 5 μg/ml (Fig. 7). Linear regression analysis gave an R2 value of 0.999, indicating that the assay is linear in this ATD concentration range.

### Immunofluorescence detection of ANRE patient CSF and sera binding to 293T-ATD cells

**Fig. 8:**
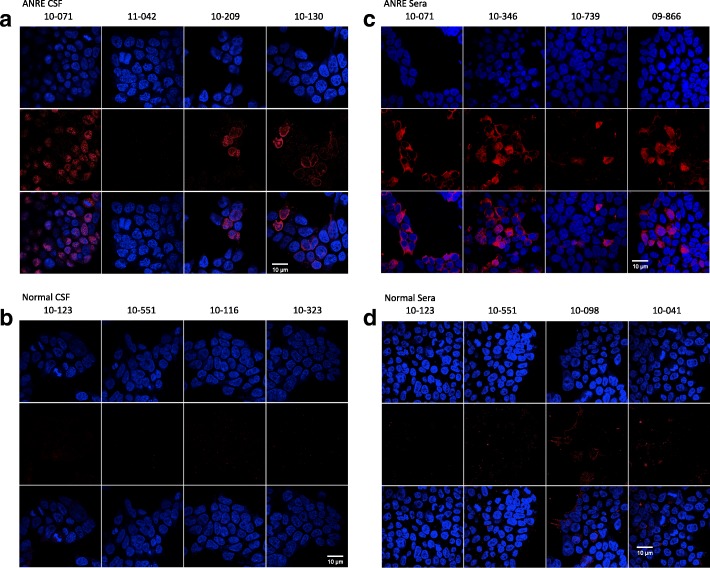
Binding of ANRE and normal human CSF and sera to 293T-ATD cells by immunofluorescence. 293T-ATD cells were stained with (**a**) ANRE patient CSF, (**b**) normal human CSF, (**c**) ANRE patient sera, and (**d**) normal human sera. Matched pairs include ANRE patient 10–071 (**a, c**) and normal subjects 10–123 and 10–551 (**d, d**). CSF were tested at 1:20 dilution; sera at 1:100. Human IgG binding is shown in red. Nuclei were stained with DAPI (blue) and the cells were visualized by confocal microscopy. Scale bars = 10 μm

We tested binding of clinical samples of ANRE and normal CSF and sera to the 293T-ATD cell line by NR1 antibodies by immunofluorescence. The samples were obtained from the clinical services at the Children’s Hospital of Philadelphia. Four ANRE and four normal human CSF (1:20) and serum samples (1:100) were tested, including a matched CSF:serum pair from ANRE patient 10–071 and two pairs from normal patients 10–123 and 10–551. Three of the ANRE CSF samples (Fig. 8a) and all four of the serum samples (Fig. 8c) gave a bright immunofluorescent signal, whereas none of the normal CSF or serum samples showed binding (Fig. 8b, c).

## Discussion

ANRE is a potentially fatal auto-immune encephalitis mediated by antibodies that bind NR1 in the hippocampus. Definitive diagnosis of ANRE requires detection of anti-NR1 IgG in patient CSF. The antigen recognized by the pathogenic IgGs in ANRE is conformational and depends on post-translational glycosylation that can only be produced in a mammalian cell. Because NMDAR over-expression can be toxic to cultured cells, the first-line clinical tests for ANRE are a CBA or ELISA that uses 293T cells transiently expressing NMDAR. The need for transfected cells to test anti-NMDAR IgG introduces variability into the assay, and limits the types of tests that can be used for ANRE diagnosis.

We developed a stable cell line that homogeneously expresses the pathogenic ATD epitope(s). The 293T-ATD cell line expresses the ATD as a fusion protein that includes the transmembrane domain of the PDGFR, which anchors the ATD to the outer plasma membrane. It also expresses a Myc tag, a 6XHIS tag, and a TEV protease site that is adjacent to the transmembrane domain. Retroviral transduction of the fusion gene, followed by FACS selection of cells recognized by the murine anti-GluN1 mAb, resulted in a population of 293T-ATD cells with uniform expression levels and a Myc-tag useful as a positive control for antigen expression. The 293T-ATD cell line specifically bound a commercial anti-GluN1 mAb, CSF from an ANRE patient, and three anti-NMDAR mAbs isolated from another ANRE patient in both flow cytometry and immunofluorescence microscopy experiments. These experiments demonstrate that pathogenic ANRE epitopes on the ATD are preserved when displayed on the outer plasma membrane. Thus, 293T-ATD cell line may be suitable for use in cell-based assays to diagnose ANRE. Expanded studies of ANRE patient IgGs will be necessary to determine whether the 293T-ATD can substitute for transiently transfected cells in clinical diagnostic testing.

Because the ATD fusion protein contained to a TEV protease site, it could be released from PBS-washed 293T-ATD cells with TEV protease, resulting in an essentially pure, intact ATD that preserved pathogenic antigens and could be used in ELISAs without additional purification. When adhered to an ELISA plate by an anti-6XHIS antibody, the ATD was specifically recognized by the commercial NR1 and human ANRE mAbs. In soluble form, the ATD demonstrated linear binding activity to a plate-adherent 5F5 anti-NMDAR mAb.

We also tested a panel of ANRE and normal patient CSF and sera. Four of five ANRE patient CSF (including the standard positive control sample shown in Fig. 4) and all four ANRE patient sera bound the cell line, whereas none of the normal samples did. Additional clinical studies of ANRE patient IgGs will be necessary to determine the spectrum of pathogenic antibodies that recognize the ATD in these assay formats. Taken together, these assays demonstrate that the soluble ATD maintains ANRE pathogenic epitopes. It is therefore potentially adaptable to a variety of non-cell-based test formats to diagnose ANRE, including ELISAs and lateral flow assays. In addition, the soluble ATD will be useful for anti-NMDAR mAb screening, epitope mapping, and affinity measurement.

Our method of expressing proteins in a membrane-tethered, cleavable form offers advantages for the production of a wide variety of proteins in mammalian cells. First, stable, high-expressing cells can be readily identified and isolated, and the Myc tag can be used as a positive control for protein expression. Isolating pure protein is straightforward, because the cells themselves provide a solid phase for separating the antigen from the culture medium, and the precise activity of the TEV protease releases essentially pure recombinant protein from the cells. The 6XHIS tag in the fusion protein can be used for additional column chromatography, if necessary, especially if large or very pure protein preps are required, and both the Myc and 6XHIS tags can be used to capture the secreted protein for solid phase binding assays. Furthermore, any protein can potentially be sorted to the outer plasma membrane by incorporating a heterologous N-terminal signal peptide [14].

## Conclusions

The 293T-ATD cell line may enable improved diagnostic tests for ANRE and studies of antibodies associated with ANRE. Ectopic expression of proteins in a tagged, cleavable form, on the outer plasma membrane of cultured mammalian cells, has the potential to expand the spectrum of antigens available for research, drug discovery, and clinical diagnosis.

## Methods

### Human subjects

CSF and patient sera were collected at the Children’s Hospital of Pennsylvania (CHOP), Philadelphia, PA, with full informed consent and protocols approved by the CHOP Institutional Review Board.

### Expression of the ATD fusion protein in 293T cells

We made a fusion gene that expresses the entire 561 amino acids of the N-terminal extracellular domain of human GluN1 (NR1) (UniProtKB - Q05586), including the amino terminal domain (ATD), followed by the Myc tag, a 6XHIS tag, a TEV protease cleavage site, and the transmembrane domain of the human platelet-derived growth factor receptor (PDGFR) (Additional file 1, Genbank Accession #) [13, 15]. The gene was synthesized and inserted into the retroviral vector, pBabe puro by Genscript (Piscataway, NJ) [16]. Amphotropic retroviruses were produced in 293T cells following standard protocols, except that X-tremeGENE 9 DNA Transfection Reagent was used (354,087, Roche, Germany), and the cells were cultured in Advanced DMEM, 1% IFS, penicillin/streptomycin (Invitrogen, Carlsbad, CA) [17].

The retroviral supernatant was used to transduce 293T cells (2.5 X10^6^ in a 10 cm dish), with 4 μg/ml polybrene (TR1003G, Thermo Fisher Scientific, Waltham, MA), for 6 h. 48 h later, cells were selected with 1 μg/ml puromycin (P9620, Sigma-Aldrich, St. Louis, MO). One week later, expressing cells were isolated by FACS staining with the murine anti-NR1 APC mAb (orb149996, Biorbyt, San Francisco, CA) on the BD FACSCanto II (Becton Dickson, Franklin Lakes, NJ). Four rounds of FACS over 4 weeks were performed to isolate a stable, homogeneous population of cells (293T-ATD).

### Flow cytometry, FACS, and immunofluorescence studies

To assess antibody binding to 293T-ATD cells by flow cytometry, cells were harvested using 0.05% trypsin, washed, and resuspended at 1 × 10^6^ cells/ml in PBS-1% BSA (A7030, Sigma-Aldrich). Primary antibodies included the Bioorbyt APC-labeled NR1 mAb at 10 μg/mL, an Alexa Fluor 488 labeled Myc tag mAb at 2 μg/mL (16–308, Millipore, Billerica, MA) three human IgG mAbs (5 μg/mL) isolated from a patient with ANRE (5F5, 2G6, 1D1, *manuscript in press*) and the 6A isotype control mAb [17]. As a secondary antibody for the human mAbs, we used a FITC-conjugated F(ab’)2 goat anti-human IgG (109–096-008, Jackson ImmunoResearch, West Grove, PA). Cells were assayed with a BD FACSCanto II (Becton Dickson, Franklin Lakes, NJ). Data were analyzed using FlowJo 8.8.7. Software (Tree Star, Ashland, OR).

For immunofluorescence studies, 293T-ATD cells were plated at 5 × 10^4^ cells/well on round Corning™ BioCoat™ 12 mm #1 German Glass Coverslips (354,087, Corning, NY) in 24 well plates. 24 h later, the cells were fixed with 4% paraformaldehyde in PBS for 10 min at room temperature, washed with PBS 0.05% Tween-20 (PBST), blocked with 10% Goat serum (Invitrogen) + 1% BSA in PBS (PBS + G + B) for 1 h at 37 °C, and then washed with PBST. Cells were incubated for one hour at room temp in PBS + G + B with one or more of the following added: a murine anti-NR1 APC mAb (orb149996, Biorbyt, San Francisco, CA)**,** an Alexa Fluor 488 conjugated anti-Myc-tag-specific mAb (16–308, Millipore) (1:250 dilution), ANRE patient or normal human CSF (1:20), ANRE patient or normal human sera (1:100), human mAbs, 5F5, 2G6, 1D1 or isotype human control mAb 8E1 (5 μg/ml). After one hour, cells were washed twice with PBST and incubated with the Alexa 568 goat anti-human IgG, 1:1000 (A21090, Thermo Fisher), secondary antibody for the human CSF or mAbs in PBS + G + B for one hour, and then the cells were washed once with PBS and once with dH_2_O. Coverslips were mounted with ProLong® Gold Antifade reagent with DAPI (P36935, Thermo Fisher) and imaged with a C2+ Nikon confocal microscope with 63×/1.3 NA oil objective; images were analyzed with ImageJ software (https://imagej.nih.gov/ij/). All immunofluorescence studies were performed at least twice.

### Mobilization of membrane-bound ATD with TEV protease

The 293T-ATD cells were plated at 2 × 10^5^ cells/well in 12 well plates. 24 h later, they were washed with PBS and then treated with 25 μg rTEV Protease (4469; R&D Systems, Minneapolis, MN) with Xpert Protease inhibitor cocktail solution (P3100–001; GenDEPOT, Barker, TX) in PBS for the indicated time period (10–40 min). The cells were pipetted up and down, transferred to Eppendorf tubes, and centrifuged at 3000 rpm for 10 min at 4 °C. The supernatant was collected and immediately dialyzed against cold PBS overnight. The protein concentration was measured using NanoDrop 1000 (Thermo Fisher) and protein was visualized on a Coomassie-stained SDS:PAGE gel.

### ATD ELISAs

To analyze the timecourse of ATD mobilization by TEV, we performed a capture assay in which we coated Black 96-well immune plates (12–566-24, Thermo Fisher) with 5 μg/mL anti HIS tag antibody (ab18184, Abcam, Cambridge, MA) overnight, washed the plates 3 times with PBST, blocked with 5% inactivated fetal bovine serum and 3% Goat serum (Invitrogen) in PBST for 1 h at 37 °C, then washed 3 times. ATD samples cleaved at the stated timepoints were added at 5 μg/mL and supernatant from un-cleaved cells was added as negative control, followed by 1 h incubation at 37 °C. The plates were washed three more times, and biotinylated human mAb 5F5 was added at 5 μg/mL (100 μl/well), and then incubated for 1 h at 37 °C. After three additional PBST washes, Streptavidin-poly-HRP conjugate at 1:2000 (Thermo Fisher) was added and incubated for 1 h at 37 °C. After three additional washes, Super Signal ELISA Femto Substrate was used for detection (Thermo Fisher). Relative luminescence values were measured using the Biotek Synergy II Microplate reader (BioTek Instruments, Winooski, VT, USA). Microsoft Excel was used to process the data.

To test binding of human NR1 antibodies to plate-adherent ATD, we added 5 μg/mL Myc antibody (C3956, Sigma-Aldrich) (100 μl/well) to Black 96-well plates (12–566-24, Thermo Fisher) overnight, washed the plates 3 times with PBST, blocked with 5% inactivated fetal bovine serum and 3% Goat serum (Invitrogen) in PBST for 1 h at 37 °C, washed 3 times, added 5 μg/mL ATD, and then incubated for 1 h at 37 °C and washed 3 more times. We added human mAbs, 5F5, 2G6, 1D1, and control isotype 6A at 5 μg/mL (100 μl/well), or 5 μg/mL anti-NR1 mAb (MAB 1586 R1JHL, Millipore), in triplicate samples, and incubated for 1 h at 37 °C. After three additional PBST washes, secondary antibodies were added, either an anti-human IgG HRP conjugate (9040–05 SouthernBiotech, Birmingham, AL) or anti-mouse IgG HRP conjugate (1010–05, Southern Biotech), at 1:2000 and incubated for 1 h at 37 °C, followed by 3 washes. Super Signal ELISA Femto Substrate was used for detection. Data was collected in the Biotek Synergy II Microplate reader and analyzed with Microsoft Excel.

To test binding of TEV-mobilized ATD to plate-adherent human IgG by ELISA, we first biotinylated the ATD using the EZ-Link™ Sulfo-NHS-Biotin kit (21,326, Thermo Fisher). We added 5 μg/mL 5F5 (100 μl/well) to Black 96-well plates (12–566-24, Thermo Fisher), incubated overnight at room temp, washed the plates 3 times with PBST, blocked the wells with 2% non-fat milk in PBST for 1 h at 37 °C, and again washed 3 times. We added triplicate serial dilutions of the biotinylated ATD (diluted in 50 μL PBS/well), and incubated for 1 h at 37 °C. After three PBST washes, Streptavidin-poly-HRP conjugate at 1:2000 (Thermo Fisher) was added and incubated for 1 h at 37 °C. Super Signal ELISA Femto Substrate was used for detection. Data was collected in the Biotek Synergy II Microplate reader and analyzed with Microsoft Excel.

## Additional file

Additional file 1: DNA and amino acid sequences of the ATD fusion protein. (DOCX 287 kb)

## Abbreviations

ANRE: Anti-NMDA receptor encephalitis
ATD: Amino terminal domain
CSF: Cerebrospinal fluid
ELISA: Enzyme linked immunosorbent assay
NMDAR: N-methyl-D-aspartate receptor
PDGF: Platelet derived growth factor
SA-HRP: Streptavidin-horseradish peroxidase
TEV: Tobacco Etch Virus
