# Supplementary material for: Membrane-bound and soluble forms of an NMDA receptor extracellular domain retain epitopes targeted in auto-immune encephalitis
Source: BMC Biotechnol. 2018 Jun 27;18:41. doi: 10.1186/s12896-018-0450-1 (PMC6020338; doi:10.1186/s12896-018-0450-1)
Supplement: Supplementary file 1 — DNA and amino acid sequences of the ATD fusion protein. (DOCX 287 kb) [file 12896_2018_450_MOESM1_ESM.pdf]

GRIN1 ATD

== Enzymes that DO NOT MAP to this sequence:

AarI	AclI	AflII	AgeI	AscI	AseI	AvrII
BbvCI	BclI	BsaI	BsiWI	BspEI	BsrGI	BstBI
BstEII	Bsu36I	ClaI	EagI	HindIII	KflI	MauBI
MfeI	MluI	MreI	NcoI	NdeI	NheI	NotI
PciI	RsrII	SalI	SgrAI	SgrDI	SpeI	XbaI

== Total Number of Hits per Enzyme:

AbsI	1	BseYI	1	EcoNI	1	PspOMI	1
Acc65I	1	BsmBI	1	**EcoRI**	**1**	PspXI	1
ApaLI	2	BspHI	1	KasI	1	SapI	1
**BamHI**	**1**	BspMI	2	KroI	1	SexAI	1
BbsI	2	BssHII	2	NarI	1	Tth111I	1
BglII	1	BssSI	3	NgoMIV	1	XhoI	2
BlpI	2	BtgZI	1	PasI	1	XmaI	1
Bpu10I	1	EarI	2	PfoI	1		

== Linear Map of Sequence:

```
          BamHI       start GRIN1
          \
      1   ggatccgccaccatgagcaccatgcgcctgctgacgctcgccctgctgttctcctgctcc     60
          cctaggcggtggtactcgtggtacgcggacgactgcgagcgggacgacaagaggacgagg
              ^    *    ^    *    ^    *    ^    *    ^    *    ^    *
1         G  S  A  T  M  S  T  M  R  L  L  T  L  A  L  L  F  S  C  S

                                                          BlpI
                                                          \
     61   gtcgcccgtgccgcgtgcgaccccaagatcgtcaacattggcgcggtgctgagcacgcgg    120
          cagcgggcacggcgcacgctggggttctagcagttgtaaccgcgccacgactcgtgcgcc
              ^    *    ^    *    ^    *    ^    *    ^    *    ^    *
1         V  A  R  A  A  C  D  P  K  I  V  N  I  G  A  V  L  S  T  R

             BssSI                                              PfoI
             \                                                  \
    121   aagcacgagcagatgttccgcgaggccgtgaaccaggccaacaagcggcacggctcctgg    180
          ttcgtgctcgtctacaaggcgctccggcacttggtccggttgttcgccgtgccgaggacc
              ^    *    ^    *    ^    *    ^    *    ^    *    ^    *
1         K  H  E  Q  M  F  R  E  A  V  N  Q  A  N  K  R  H  G  S  W


    181   aagattcagctcaatgccacctccgtcacgcacaagcccaacgccatccagatggctctg    240
          ttctaagtcgagttacggtggaggcagtgcgtgttcgggttgcggtaggtctaccgagac
              ^    *    ^    *    ^    *    ^    *    ^    *    ^    *
1         K  I  Q  L  N  A  T  S  V  T  H  K  P  N  A  I  Q  M  A  L
```

```
    241   tcggtgtgcgaggacctcatctccagccaggtctacgccatcctagttagccatccacct    300
          agccacacgctcctggagtagaggtcggtccagatgcggtaggatcaatcggtaggtgga
              ^    *    ^    *    ^    *    ^    *    ^    *    ^    *
1         S  V  C  E  D  L  I  S  S  Q  V  Y  A  I  L  V  S  H  P  P

                                                    NgoMIV
                                                    KroI
                                                    \
    301   acccccaacgaccacttcactcccacccctgtctcctacacagccggcttctaccgcata    360
          tgggggttgctggtgaagtgagggtggggacagaggatgtgtcggccgaagatggcgtat
              ^    *    ^    *    ^    *    ^    *    ^    *    ^    *
1         T  P  N  D  H  F  T  P  T  P  V  S  Y  T  A  G  F  Y  R  I

                   BseYI                                    Bpu10I
                   \                                        \
    361   cccgtgctggggctgaccacccgcatgtccatctactcggacaagagcatccacctgagc    420
          gggcacgaccccgactggtgggcgtacaggtagatgagcctgttctcgtaggtggactcg
              ^    *    ^    *    ^    *    ^    *    ^    *    ^    *
1         P  V  L  G  L  T  T  R  M  S  I  Y  S  D  K  S  I  H  L  S

    421   ttcctgcgcaccgtgccgccctactcccaccagtccagcgtgtggtttgagatgatgcgt    480
          aaggacgcgtggcacggcgggatgagggtggtcaggtcgcacaccaaactctactacgca
              ^    *    ^    *    ^    *    ^    *    ^    *    ^    *
1         F  L  R  T  V  P  P  Y  S  H  Q  S  S  V  W  F  E  M  M  R

                                                    BssSI
                                                    \
```

```
     481   gtctacagctggaaccacatcatcctgctggtcagcgacgaccacgagggccgggcggct    540
           cagatgtcgaccttggtgtagtaggacgaccagtcgctgctggtgctcccggcccgccga
               ^    *    ^    *    ^    *    ^    *    ^    *    ^    *
1          V  Y  S  W  N  H  I  I  L  L  V  S  D  D  H  E  G  R  A  A

                     BsmBI
                     \
     541   cagaaacgcctggagacgctgctggaggagcgtgagtccaaggcagagaaggtgctgcag    600
           gtctttgcggacctctgcgacgacctcctcgcactcaggttccgtctcttccacgacgtc
               ^    *    ^    *    ^    *    ^    *    ^    *    ^    *
1          Q  K  R  L  E  T  L  L  E  E  R  E  S  K  A  E  K  V  L  Q

                 PasI                                                  XmaI
                 \                                                     \
     601   tttgacccagggaccaagaacgtgacggccctgctgatggaggcgaaagagctggaggcc    660
           aaactgggtccctggttcttgcactgccgggacgactacctccgctttctcgacctccgg
               ^    *    ^    *    ^    *    ^    *    ^    *    ^    *
1          F  D  P  G  T  K  N  V  T  A  L  L  M  E  A  K  E  L  E  A

     661   cgggtcatcatcctttctgccagcgaggacgatgctgccactgtataccgcgcagccgcg    720
           gcccagtagtaggaaagacggtcgctcctgctacgacggtgacatatggcgcgtcggcgc
               ^    *    ^    *    ^    *    ^    *    ^    *    ^    *
1          R  V  I  I  L  S  A  S  E  D  D  A  A  T  V  Y  R  A  A  A

                       BtgZI                               BglII
                       \                                   \
     721   atgctgaacatgacgggctccgggtacgtgtggctggtcggcgagcgcgagatctcgggg    780
           tacgacttgtactgcccgaggcccatgcacaccgaccagccgctcgcgctctagagcccc
               ^    *    ^    *    ^    *    ^    *    ^    *    ^    *
```

```
1         M  L  N  M  T  G  S  G  Y  V  W  L  V  G  E  R  E  I  S  G

    781   aacgccctgcgctacgccccagacggcatcctcgggctgcagctcatcaacggcaagaac    840
          ttgcgggacgcgatgcggggtctgccgtaggagcccgacgtcgagtagttgccgttcttg
              ^    *    ^    *    ^    *    ^    *    ^    *    ^    *
1         N  A  L  R  Y  A  P  D  G  I  L  G  L  Q  L  I  N  G  K  N

                                                         BssSI
                                                      ApaLI       XhoI
                                                      \  \        \
    841   gagtcggcccacatcagcgacgccgtgggcgtggtggcccaggccgtgcacgagctcctc    900
          ctcagccgggtgtagtcgctgcggcacccgcaccaccgggtccggcacgtgctcgaggag
              ^    *    ^    *    ^    *    ^    *    ^    *    ^    *
1         E  S  A  H  I  S  D  A  V  G  V  V  A  Q  A  V  H  E  L  L

          EcoNI
          \
    901   gagaaggagaacatcaccgacccgccgcggggctgcgtgggcaacaccaacatctggaag    960
          ctcttcctcttgtagtggctgggcggcgccccgacgcacccgttgtggttgtagaccttc
              ^    *    ^    *    ^    *    ^    *    ^    *    ^    *
1         E  K  E  N  I  T  D  P  P  R  G  C  V  G  N  T  N  I  W  K

                         EarI
             BbsI        SapI       BbsI
             \           \          \
    961   accgggccgctcttcaagagagtgctgatgtcttccaagtatgcggatggggtgactggt   1020
          tggcccggcgagaagttctctcacgactacagaaggttcatacgcctaccccactgacca
              ^    *    ^    *    ^    *    ^    *    ^    *    ^    *
1         T  G  P  L  F  K  R  V  L  M  S  S  K  Y  A  D  G  V  T  G
```

```
                                                           BspHI
                                                           \
   1021   cgcgtggagttcaatgaggatggggaccggaagttcgccaactacagcatcatgaacctg   1080
          gcgcacctcaagttactcctacccctggccttcaagcggttgatgtcgtagtacttggac
              ^    *    ^    *    ^    *    ^    *    ^    *    ^    *
1         R  V  E  F  N  E  D  G  D  R  K  F  A  N  Y  S  I  M  N  L

              BspMI
              \
   1081   cagaaccgcaagctggtgcaagtgggcatctacaatggcacccacgtcatccctaatgac   1140
          gtcttggcgttcgaccacgttcacccgtagatgttaccgtgggtgcagtagggattactg
              ^    *    ^    *    ^    *    ^    *    ^    *    ^    *
1         Q  N  R  K  L  V  Q  V  G  I  Y  N  G  T  H  V  I  P  N  D

                                               XhoI
                                               PspXI
                                               AbsI  Acc65I
                                               \     \
   1141   aggaagatcatctggccaggcggagagacagagaagcctcgagggtaccagatgtccacc   1200
          tccttctagtagaccggtccgcctctctgtctcttcggagctcccatggtctacaggtgg
              ^    *    ^    *    ^    *    ^    *    ^    *    ^    *
1         R  K  I  I  W  P  G  G  E  T  E  K  P  R  G  Y  Q  M  S  T


   1201   agactgaagattgtgacgatccaccaggagcccttcgtgtacgtcaagcccacgctgagt   1260
          tctgacttctaacactgctaggtggtcctcgggaagcacatgcagttcgggtgcgactca
              ^    *    ^    *    ^    *    ^    *    ^    *    ^    *
1         R  L  K  I  V  T  I  H  Q  E  P  F  V  Y  V  K  P  T  L  S
```

```
                                                       Tth111I
                                                       \
   1261   gatgggacatgcaaggaggagttcacagtcaacggcgacccagtcaagaaggtgatctgc   1320
          ctaccctgtacgttcctcctcaagtgtcagttgccgctgggtcagttcttccactagacg
              ^    *    ^    *    ^    *    ^    *    ^    *    ^    *
1         D  G  T  C  K  E  E  F  T  V  N  G  D  P  V  K  K  V  I  C

             PspOMI
             \
   1321   accgggcccaacgacacgtcgccgggcagcccccgccacacggtgcctcagtgttgctac   1380
          tggcccgggttgctgtgcagcggcccgtcgggggcggtgtgccacggagtcacaacgatg
              ^    *    ^    *    ^    *    ^    *    ^    *    ^    *
1         T  G  P  N  D  T  S  P  G  S  P  R  H  T  V  P  Q  C  C  Y

                                BspMI                              ApaLI
                                \                                  \
   1381   ggcttttgcatcgacctgctcatcaagctggcacggaccatgaacttcacctacgaggtg   1440
          ccgaaaacgtagctggacgagtagttcgaccgtgcctggtacttgaagtggatgctccac
              ^    *    ^    *    ^    *    ^    *    ^    *    ^    *
1         G  F  C  I  D  L  L  I  K  L  A  R  T  M  N  F  T  Y  E  V

           SexAI
           \
   1441   cacctggtggcagatggcaagttcggcacacaggagcgggtgaacaacagcaacaagaag   1500
          gtggaccaccgtctaccgttcaagccgtgtgtcctcgcccacttgttgtcgttgttcttc
              ^    *    ^    *    ^    *    ^    *    ^    *    ^    *
1         H  L  V  A  D  G  K  F  G  T  Q  E  R  V  N  N  S  N  K  K

                                                                NarI
                                     BlpI                      KasI
                                     \                         \\
```

```
1501   gagtggaatgggatgatgggcgagctgctcagcgggcaggcagacatgatcgtggcgccg   1560
       ctcaccttaccctactacccgctcgacgagtcgcccgtccgtctgtactagcaccgcggc
           ^    *    ^    *    ^    *    ^    *    ^    *    ^    *
1      E  W  N  G  M  M  G  E  L  L  S  G  Q  A  D  M  I  V  A  P

                                BssHII
                             BssHII
                             \ \
1561   ctaaccataaacaacgagcgcgcgcagtacatcgagttttccaagcccttcaagtaccag   1620
       gattggtatttgttgctcgcgcgcgtcatgtagctcaaaaggttcgggaagttcatggtc
           ^    *    ^    *    ^    *    ^    *    ^    *    ^    *
1      L  T  I  N  N  E  R  A  Q  Y  I  E  F  S  K  P  F  K  Y  Q

1621   ggcctgactattctggtcaagaaggagattccccggagcacgctggactcgttcatgcag   1680
       ccggactgataagaccagttcttcctctaaggggcctcgtgcgacctgagcaagtacgtc
           ^    *    ^    *    ^    *    ^    *    ^    *    ^    *
1      G  L  T  I  L  V  K  K  E  I  P  R  S  T  L  D  S  F  M  Q

                  myc tag         EarI                 HIS TAG
                                   \
1681   ccgttccagagcacagaacaaaaactcatctcagaagaggatctgcatcatcaccatcac   1740 *T modified
       ggcaaggtctcgtgtcttgtttttgagtagagtcttctcctagacgtagtagtggtagtg        by Furukawa
           ^    *    ^    *    ^    *    ^    *    ^    *    ^    *
1      P  F  Q  S  T* E  Q  K  L  I  S  E  E  D  L  H  H  H  H  H

          AcTEV protease site   Asp PDGF TM domain>>>

1741   cacgaaaacctgtattttcagggcggcaacgctgtgggccaggacacgcaggaggtcatc   1800
       gtgcttttggacataaaagtcccgccgttgcgacacccggtcctgtgcgtcctccagtag
```

```
              ^    *    ^    *    ^    *    ^    *    ^    *    ^    *
1         H  E  N  L  Y  F  Q  G  G  N  A  V  G  Q  D  T  Q  E  V  I


   1801   gtggtgccacactccttgccctttaaggtggtggtgatctcagccatcctggccctggtg   1860
          caccacggtgtgaggaacgggaaattccaccaccactagagtcggtaggaccgggaccac
              ^    *    ^    *    ^    *    ^    *    ^    *    ^    *
1         V  V  P  H  S  L  P  F  K  V  V  V  I  S  A  I  L  A  L  V


   1861   gtgctcaccatcatctcccttatcatcctcatcatgctttggcagaagaagccacgttag   1920
          cacgagtggtagtagagggaatagtaggagtagtacgaaaccgtcttcttcggtgcaatc
              ^    *    ^    *    ^    *    ^    *    ^    *    ^    *
1         V  L  T  I  I  S  L  I  I  L  I  M  L  W  Q  K  K  P  R  *

          EcoRI
          \
   1921   gaattc                                                         1980
          cttaag
              ^    *    ^    *    ^    *    ^    *    ^    *    ^    *
1         E  F

 ==--------------------------  End of Analysis  --------------------------==
```

>GRIN1 ATD fusion protein
GGATCCGCCACCATGAGCACCATGCGCCTGCTGACGCTCGCCCTGCTGTTCTCCTGCTCCGTCGCCCGTG
CCGCGTGCGACCCCAAGATCGTCAACATTGGCGCGGTGCTGAGCACGCGGAAGCACGAGCAGATGTTCCG
CGAGGCCGTGAACCAGGCCAACAAGCGGCACGGCTCCTGGAAGATTCAGCTCAATGCCACCTCCGTCACG
CACAAGCCCAACGCCATCCAGATGGCTCTGTCGGTGTGCGAGGACCTCATCTCCAGCCAGGTCTACGCCA
TCCTAGTTAGCCATCCACCTACCCCCAACGACCACTTCACTCCCACCCCTGTCTCCTACACAGCCGGCTT
CTACCGCATACCCGTGCTGGGGCTGACCACCCGCATGTCCATCTACTCGGACAAGAGCATCCACCTGAGC
TTCCTGCGCACCGTGCCGCCCTACTCCCACCAGTCCAGCGTGTGGTTTGAGATGATGCGTGTCTACAGCT
GGAACCACATCATCCTGCTGGTCAGCGACGACCACGAGGGCCGGGCGGCTCAGAAACGCCTGGAGACGCT
GCTGGAGGAGCGTGAGTCCAAGGCAGAGAAGGTGCTGCAGTTTGACCCAGGGACCAAGAACGTGACGGCC
CTGCTGATGGAGGCGAAAGAGCTGGAGGCCCGGGTCATCATCCTTTCTGCCAGCGAGGACGATGCTGCCA
CTGTATACCGCGCAGCCGCGATGCTGAACATGACGGGCTCCGGGTACGTGTGGCTGGTCGGCGAGCGCGA
GATCTCGGGGAACGCCCTGCGCTACGCCCCAGACGGCATCCTCGGGCTGCAGCTCATCAACGGCAAGAAC
GAGTCGGCCCACATCAGCGACGCCGTGGGCGTGGTGGCCCAGGCCGTGCACGAGCTCCTCGAGAAGGAGA
ACATCACCGACCCGCCGCGGGGCTGCGTGGGCAACACCAACATCTGGAAGACCGGGCCGCTCTTCAAGAG
AGTGCTGATGTCTTCCAAGTATGCGGATGGGGTGACTGGTCGCGTGGAGTTCAATGAGGATGGGGACCGG
AAGTTCGCCAACTACAGCATCATGAACCTGCAGAACCGCAAGCTGGTGCAAGTGGGCATCTACAATGGCA
CCCACGTCATCCCTAATGACAGGAAGATCATCTGGCCAGGCGGAGAGACAGAGAAGCCTCGAGGGTACCA
GATGTCCACCAGACTGAAGATTGTGACGATCCACCAGGAGCCCTTCGTGTACGTCAAGCCCACGCTGAGT
GATGGGACATGCAAGGAGGAGTTCACAGTCAACGGCGACCCAGTCAAGAAGGTGATCTGCACCGGGCCCA
ACGACACGTCGCCGGGCAGCCCCCGCCACACGGTGCCTCAGTGTTGCTACGGCTTTTGCATCGACCTGCT
CATCAAGCTGGCACGGACCATGAACTTCACCTACGAGGTGCACCTGGTGGCAGATGGCAAGTTCGGCACA
CAGGAGCGGGTGAACAACAGCAACAAGAAGGAGTGGAATGGGATGATGGGCGAGCTGCTCAGCGGGCAGG
CAGACATGATCGTGGCGCCGCTAACCATAAACAACGAGCGCGCGCAGTACATCGAGTTTTCCAAGCCCTT
CAAGTACCAGGGCCTGACTATTCTGGTCAAGAAGGAGATTCCCCGGAGCACGCTGGACTCGTTCATGCAG
CCGTTCCAGAGCACAGAACAAAAACTCATCTCAGAAGAGGATCTGCATCATCACCATCACCACGAAAACC
TGTATTTTCAGGGCGGCAACGCTGTGGGCCAGGACACGCAGGAGGTCATCGTGGTGCCACACTCCTTGCC
CTTTAAGGTGGTGGTGATCTCAGCCATCCTGGCCCTGGTGGTGCTCACCATCATCTCCCTTATCATCCTC
ATCATGCTTTGGCAGAAGAAGCCACGTTAGGAATTC

>GRIN1 ATD FUSION PROTEIN amino acid sequence
MSTMRLLTLALLFSCSVARAACDPKIVNIGAVLSTRKHEQMFREAVNQANKRHGSWKIQLNATSVTHKPNAIQMALSVCEDLISSQVYA
ILVSHPPTPNDHFTPTPVSYTAGFYRIPVLGLTTRMSIYSDKSIHLSFLRTVPPYSHQSSVWFEMMRVYSWNHIILLVSDDHEGRAAQK
RLETLLEERESKAEKVLQFDPGTKNVTALLMEAKELEARVIILSASEDDAATVYRAAAMLNMTGSGYVWLVGEREISGNALRYAPDGIL
GLQLINGKNESAHISDAVGVVAQAVHELLEKENITDPPRGCVGNTNIWKTGPLFKRVLMSSKYADGVTGRVEFNEDGDRKFANYSIMNL
QNRKLVQVGIYNGTHVIPNDRKIIWPGGETEKPRGYQMSTRLKIVTIHQEPFVYVKPTLSDGTCKEEFTVNGDPVKKVICTGPNDTSPG
SPRHTVPQCCYGFCIDLLIKLARTMNFTYEVHLVADGKFGTQERVNNSNKKEWNGMMGELLSGQADMIVAPLTINNERAQYIEFSKPFK
YQGLTILVKKEIPRSTLDSFMQPFQSTEQKLISEEDLHHHHHHENLYFQGGNAVGQDTQEVIVVPHSLPFKVVVISAILALVVLTIISL
IILIMLWQKKPR
